# Artificial intelligence in variant calling: a review

**DOI:** 10.3389/fbinf.2025.1574359

**Published:** 2025-04-23

**Authors:** Omar Abdelwahab, Davoud Torkamaneh

**Affiliations:** ^1^ Département de Phytologie, Université Laval, Québec City, QC, Canada; ^2^ Institut de Biologie Intégrative et des Systèmes (IBIS), Université Laval, Québec City, QC, Canada; ^3^ Centre de recherche et d’innovation sur les végétaux (CRIV), Université Laval, Québec City, QC, Canada; ^4^ Institut intelligence et données (IID), Université Laval, Québec City, QC, Canada

**Keywords:** variant calling, artificial intelligence, deep learning, genomics, machine learning (ML)

## Abstract

Artificial intelligence (AI) has revolutionized numerous fields, including genomics, where it has significantly impacted variant calling, a crucial process in genomic analysis. Variant calling involves the detection of genetic variants such as single nucleotide polymorphisms (SNPs), insertions/deletions (InDels), and structural variants from high-throughput sequencing data. Traditionally, statistical approaches have dominated this task, but the advent of AI led to the development of sophisticated tools that promise higher accuracy, efficiency, and scalability. This review explores the state-of-the-art AI-based variant calling tools, including DeepVariant, DNAscope, DeepTrio, Clair, Clairvoyante, Medaka, and HELLO. We discuss their underlying methodologies, strengths, limitations, and performance metrics across different sequencing technologies, alongside their computational requirements, focusing primarily on SNP and InDel detection. By comparing these AI-driven techniques with conventional methods, we highlight the transformative advancements AI has introduced and its potential to further enhance genomic research.

## Introduction

Over the past few decades, the rapid advancement of next-generation sequencing (NGS) technologies has revolutionized the field of genomics. This technological evolution has facilitated the generation of vast amounts of data, which have greatly expanded our understanding of various biological processes, diseases, and genetic disorders (Bamshad et al., 2012; Koboldt et al., 2012; Timothy et al., 2013; Walter et al., 2015). Among the most common and impactful applications of NGS are whole genome sequencing (WGS) and whole exome sequencing (WES), which have become essential tools for dissecting genetic basis of different genetic conditions and development of diagnostic tools and guiding therapeutic decisions (Bodian et al., 2015; Willig et al., 2015; Smith et al., 2016; Schon et al., 2021). However, the sheer volume of data produced by these technologies presents significant challenges in terms of data storage, management and analysis.

To address these challenges, increasingly sophisticated computational pipelines and algorithms have been developed (McKenna et al., 2010; Garrison and Marth, 2012; Rimmer et al., 2014; Danecek et al., 2021; Helal et al., 2024), with a key focus on variant calling—the identification and characterization of genetic variants, such as single nucleotide polymorphisms (SNPs), insertions/deletions (InDels), and structural variants. Variant calling is a multi-step process in genomic analysis that involves sequencing, mapping, calling, and refinement. First, DNA or RNA is sequenced to generate raw nucleotide reads, which are then quality-checked. Next, these reads are mapped to a reference genome/transcriptome using alignment tools, producing a sequence alignment map that indicates where each read matches the reference. Variant calling tools then analyze the aligned reads to detect genetic variations which are recorded in variant call format (VCF) files. Finally, refinement steps, such as filtering to remove false positives, ensure high-confidence results for downstream analysis (Koboldt, 2020). Variant calling is a fundamental step in genomic studies, with applications ranging from population genetics to disease research and personalized medicine. It provides valuable insights into genetic diversity, disease-associated mutations, and individual genetic profiles (Maher et al., 2013; Lu et al., 2014; Witte et al., 2014; Bean and Hegde, 2016).

Historically, variant calling has relied on statistical methods to detect and interpret genomic variations that were extensively evaluated and reviewed in previous studies (Liu et al., 2013; Xu, 2018; Cameron et al., 2019; Pei et al., 2021; Helal et al., 2024; Joe et al., 2024). However, the advent of artificial intelligence (AI) has introduced a new generation of tools and pipelines that offer improved accuracy, efficiency, and scalability. In this review, we delve into the current state-of-the-art AI-based variant calling tools, examining their methodologies, strengths and limitations. We also provide a comparative analysis of their performance against conventional pipelines and discuss the broader implications of these advancements for the future of genomics.

## AI-based variant callers

AI-based variant calling tools represent a cutting-edge approach in genomic research, leveraging machine learning (ML) and deep learning (DL) algorithms to improve the accuracy and efficiency of detecting genetic variants (Hall et al., 2024). Unlike traditional rule-based tools, these AI models are trained on large-scale genomic datasets to identify subtle patterns and reduce false-positive and false-negative rates. They can handle complex genomic regions, including repetitive or highly variable areas, where conventional methods often struggle (Junjun et al., 2024). Below is an overview of the most widely used AI-based variant calling tools, focusing on their unique methodologies and features.

### DeepVariant

Developed by Google Health (The Value of Genomic Analysis - Google Health, 2022), DeepVariant is an open-source DL-based variant caller that uses deep convolutional neural networks (CNNs) to analyze pileup image tensors of aligned reads, outputting detected variants with high accuracy (Poplin et al., 2018). Initially designed for short-read data, it now supports long-read technologies such as PacBio High-Fidelity (HiFi) and Oxford nanopore technology (ONT) (Wenger et al., 2019; Shafin et al., 2021). One of the major strengths of DeepVariant is its ability to automatically produce filtered variants directly, eliminating the need for post-variant calling refinement. This feature, combined with its high accuracy compared to other tools (including SAMTools, Strelka, GATK, FreeBayes, and 16 GT) (Poplin et al., 2018), made it a preferred choice for large-scale genomic studies such as the UK Biobank WES consortium (500k individuals) (Szustakowski et al., 2021). However, the high computational cost associated with DeepVariant, despite its compatibility with both GPU and CPU, is a significant drawback (Abdelwahab et al., 2023).

### DeepTrio

DeepTrio (Kolesnikov et al., 2021) is an advanced DL-based variant caller developed by Google Health as an extension of DeepVariant, designed specifically for analyzing genomic data from family trios, typically consisting of a child and their two parents. Building upon the foundation of DeepVariant, DeepTrio leverages deep CNNs to jointly analyze sequencing data from all three family members, enhancing the accuracy of variant detection by incorporating familial context. This approach allows DeepTrio to effectively weigh sequencing errors, mapping inaccuracies, and *de novo* mutation directly from the sequence data (Kolesnikov et al., 2021), leading to improved variant calling performance, especially in challenging genomic regions and lower sequencing coverages. DeepTrio’s performance surpasses that of many traditional and non-trio variant calling methods, such as GATK (McKenna et al., 2010), Strelka (Kim et al., 2018), and FreeBayes (Garrison and Marth, 2012), by maintaining high accuracy across different sequencing technologies and coverage levels.

### DNAscope

Developed by Sentieon, DNAscope is optimized for efficiency and computational speed (Freed et al., 2022a). It was originally developed for short-read sequencing and was later adapted for PacBio HiFi data for the PrecisionFDA challenge (Freed et al., 2022b; Olson et al., 2022). Recently, it has been extended to handle ONT data (DNAscope LongRead Nanopore Pipeline - Sentieon, 2025). DNAscope, which was introduced after DNAseq (Sentieon’s GATK-matching germline variant calling pipeline) (Glusman et al., 2019), demonstrated very high SNP and InDel accuracy by combining GATK’s HaplotypeCaller with an AI-based genotyping trained model (Freed et al., 2022a). These algorithms allow DNAscope to accurately identify and classify variations with high sensitivity and specificity. One key advantage of DNAscope is its ability to process large amounts of data quickly and accurately, without the need to set filtering thresholds manually. DNAscope achieved a significant reduction in computational cost compared to other variant callers, such as DeepVariant and GATK, by reducing the memory overhead and leveraging multi-threaded processing, which leads to faster runtimes without compromising accuracy (Freed et al., 2022a). DNAscope has demonstrated strong performance in various benchmarking studies, particularly in detecting SNPs and small InDels across diverse datasets (Pei et al., 2021; Li C. et al., 2022).

It is important to note that the methodology of DNAscope relies on ML enhancements rather than DL architectures. Even though ML is widely regarded as a subset of AI (Das et al., 2015; Chhaya et al., 2020; Helm et al., 2020), DNAscope’s approach does not leverage DL techniques that are characteristic of modern AI-driven tools. Furthermore, as noted in the ‘Resource Requirements’ section, DNAscope does not require GPU acceleration, a common feature of DL-based AI models. These distinctions clarify DNAscope’s classification as an ML-assisted AI tool rather than a DL-based AI model.

### Clair, Clair3, and Clairvoyante

Clair (Luo et al., 2020) and Clair3 (Zheng et al., 2022) are DL-based variant callers that specialize in genomic variant detection from both short-read and long-read sequencing data. Developed as a successor to Clairvoyante (Luo et al., 2019), Clair builds on its predecessor’s foundation, incorporating advanced neural networks to achieve improved accuracy in calling SNPs and InDels. Both Clair and Clairvoyante utilize CNNs to process genomic data, leveraging DL techniques to capture complex patterns in sequencing reads and produce accurate variant calls. Clairvoyante had some drawbacks; for instance, one major drawback was the inaccuracy in the calling of multi-allelic variants (Luo et al., 2019; Luo et al., 2020; Zheng et al., 2022). Clair extends Clairvoyante’s variant calling capabilities by further optimizing the model’s architecture and training it on more diverse data, improving its performance, especially on long-read sequencing data. For example, Clair3 runs faster than any of the other state-of-the-art variant callers and achieves better performance, especially at lower coverage, which are traditionally more prone to errors (Zheng et al., 2022).

### Medaka

Medaka (Nanoporetech/medaka, 2018) is specifically designed for analyzing long-read sequencing data generated by ONT. It employs neural networks to perform haploid-aware variant calling, which allows it to account for the inherent error rates associated with ONT sequencing, while still producing accurate variant calls for SNPs and InDels. Medaka’s variant calling pipeline typically follows after base-calling and alignment. The tool refines the mapped reads and produces high-quality consensus sequences using DL models specifically optimized for ONT data (Nanoporetech/medaka, 2018). One of Medaka’s core strengths is its ability to correct sequencing errors common in long-read technologies by training on these datasets. This results in more reliable variant calls, particularly in complex genomic regions that are often challenging for short-read-based methods. While Medaka is particularly effective at detecting nucleotide variants, its accuracy for larger structural variants may not be as high as that of tools specifically designed for structural variant calling (Nanoporetech/medaka, 2018). The computational cost of running Medaka is moderate and extremely faster than other early tools tailored for ONT data (e.g., Nanopolish (Simpson et al., 2017; Jts/nanopolish, 2017)).

### HELLO

Hybrid and stand-alone Estimation of smaLL genOmic variants (HELLO) (Ramachandran et al., 2021) is an open-source variant caller designed to address the challenges of detecting genomic variants of both SNPs and InDels from Illumina, PacBio, and hybrid data of both Illumina and PacBio. HELLO employs a deep neural network (DNN) architecture that explicitly models the fundamental units of sequencing data, such as reads and alleles. The DNNs used in HELLO are smaller and more efficient compared to those used in DeepVariant, allowing for faster execution and reduced computational resource requirements. It introduces operations to compare allelic evidence and uses probabilistic reasoning to produce variant calls. The pipeline involves sorting and aligning reads, with specific processes for short reads and PacBio reads. For short reads, InDel realignment is performed to ensure consistent representation of InDels, while for PacBio reads, haplotag sorting and alignment to reference and alternative alleles are conducted.

## Performance comparison

AI-based variant calling tools have set a new benchmark for accuracy in detecting genetic variants across various sequencing platforms, leveraging DL architectures like CNNs and DNNs to interpret sequencing data with more nuance than traditional statistical approaches (Poplin et al., 2018; Glusman et al., 2019; Ramachandran et al., 2021; Barbitoff et al., 2022; Freed et al., 2022a; Olson et al., 2022; Wagner et al., 2022). Despite their shared goal of precision, these tools perform differently across sequencing technologies and variant types (Table 1). DeepVariant consistently delivers high SNP and InDel accuracy across Illumina and PacBio HiFi data, reaching SNP F1 scores of 99.9%, which makes it highly versatile (Poplin et al., 2018; Shafin et al., 2021; Olson et al., 2022). However, its accuracy decreases when processing ONT data, especially for InDels, due to ONT’s higher base-calling error rates, with accuracy dropping to 76.8% for these variants (Shafin et al., 2021). Nonetheless, DeepVariant remains a preferred choice for large-scale projects, like the UK Biobank WES consortium, due to its capacity for accurate, filtered variant calls without post-processing (Szustakowski et al., 2021). DeepTrio extends DeepVariant’s utility by integrating familial data from trios, allowing it to better detect variants, particularly *de novo* mutations and those in regions of low coverage. This family-based approach enables DeepTrio to achieve slightly better accuracy than DeepVariant, with SNP detection accuracy maintaining high levels at 99.8% for Illumina and 99.9% for PacBio HiFi, making it ideal for studies of rare genetic diseases and complex inheritance (Kolesnikov et al., 2021; Brand et al., 2024). While DNAscope, a commercial tool, also offers competitive accuracy, particularly for SNPs, it does so with significantly reduced computational overhead compared to DeepVariant and DeepTrio, making it suitable for high-throughput projects requiring rapid turnaround times (Freed et al., 2017; Freed et al., 2022a; Glusman et al., 2019). DNAscope’s performance on PacBio HiFi matches other leading tools, with 99.9% SNP and 99.5% InDel accuracy, further supporting its use in time-sensitive clinical settings (Freed et al., 2022b). Clair3, the latest in the Clair series, provides robust support across all sequencing platforms, particularly excelling in long-read data with PacBio HiFi and ONT, reaching 99.9% SNP accuracy and notable InDel accuracy even at lower coverage. This, combined with moderate computational needs, makes Clair3 versatile for labs with diverse sequencing platforms (Altshuler et al., 2012; Walter et al., 2015; Luo et al., 2020; Zheng et al., 2022). Medaka, meanwhile, focuses on ONT long-read sequencing and is highly reliable for SNP detection at 99.0%, though it may face challenges with larger structural variants. Its strength lies in refining ONT-specific errors, which makes it valuable for ONT-specific projects, especially with high-coverage data (Nanoporetech/medaka, 2018). Nonetheless, Medaka recommends using Clair3 for diploid variant calling as it achieves higher accuracy and has better computational performance (Nanoporetech/medaka, 2018). However, Medaka still achieves comparable accuracies across various studies (Kuno et al., 2022; Zheng et al., 2022). Lastly, HELLO stands out for its support of both Illumina and PacBio data, excelling in hybrid settings where it reaches near-perfect SNP accuracy of 99.9% and significantly reduces InDel errors, particularly in combined Illumina-PacBio data (Ramachandran et al., 2021). This makes HELLO a strong option for studies requiring the integration of multiple sequencing technologies (Hassan et al., 2023; Zeibich et al., 2023). Moreover, it is notable to mention that HELLO outperforms DeepVariant using PacBio data using any given coverage (Ramachandran et al., 2021). In summary, while all these tools achieve high SNP detection accuracy, their InDel calling performance varies significantly for long-read technologies like ONT, with DeepVariant and Clair3 leading in multi-platform accuracy, and DNAscope and HELLO noted for efficiency and hybrid capabilities, respectively. To provide a comprehensive perspective, Table 1 also includes conventional variant calling methods, GATK, BCFTools, and FreeBayes, alongside their performance metrics, which serves as a reference to better contextualize the advancements and performance improvements introduced by the AI-based variant calling tools.

**TABLE 1:** F1 scores for SNPs and InDels, called from different sequencing technologies, and corresponding coverages for each variant caller.

Tool	Sequencing technology	SNP F1 score (%)	InDel F1 score (%)	Reported coverage (X)
DeepVariant	Illumina	99.9	99.6	30
	PacBio HiFi	99.9	99.2	30
	ONT	99.8	76.8	90
DeepTrio	Illumina	99.8	99.7	35
	PacBio HiFi	99.9	99.5	35
DNAscope	Illumina	99.5	>99	30
	PacBio HiFi	99.9	99.5	30
Clair	Illumina	99.9	99.6	52
	PacBio HiFi	99.9	99.3	33
Clair3	ONT	99.3	73.17	20
Medaka	ONT	99	73.2	30 (SNPs), 50 (InDel)
HELLO	Illumina	99.6	99.5	50
	PacBio	99.9	99.7	60
HELLO (Hybrid)	Illumina + PacBio	99.9	99.8	50 (Illumina), 60 (PacBio)
GATK	Illumina	99.2	97.3	26
	PacBio	99.5	77.7	40
FreeBayes	Illumina	98	92.7	26
BCFTools	Illumina	95.7	81.2	12
	PacBio HiFi	99.3	84.9	40
	ONT	90.9	0	80

Note: The InDel F1 score of 0.0 for BCFTools on ONT data reflects experimental results reported by Abdelwahab et al., where BCFTools failed to detect any InDels, resulting in both precision and recall values of zero.

It is important to note that certain tools were not assessed for specific sequencing technologies due to their design focus and the availability of performance evaluations. DeepTrio, for example, is designed specifically for trio-based analysis and has been optimized for Illumina and PacBio HiFi data, with no published evaluations on ONT data. DNAscope, while recently adopted for ONT data, has not yet been included in comparative studies or benchmarks for this platform. HELLO is optimized for hybrid sequencing approaches (Illumina + PacBio) and currently does not support ONT-based variant calling. Conversely, Medaka is specifically designed for ONT data and is not optimized for Illumina or PacBio HiFi sequencing.

## Computational resource requirements

The computational resource demands of AI-based variant callers vary considerably, influenced by the sequencing platform, dataset size, and tool architecture, with some tools necessitating substantial resources, particularly for large-scale or long-read sequencing projects (Table 2). DeepVariant is among the more resource-intensive options; for a typical 30x coverage of human whole genome sequence (WGS) using Illumina data, it requires around 5 h on a multi-core CPU system (e.g., m5.24xlarge, 96 vCPUs), but this time can be reduced to approximately 8 min when utilizing a high-performance GPU system like an NVIDIA DGX station (Deepvariant, 2023). The resource requirements escalate further when processing large population or long-read data, as DeepVariant demands substantial RAM and CPU resources, making it better suited for cloud-based or high-performance computing (HPC) environments, which may be inaccessible to smaller labs with limited infrastructure. DeepTrio, which builds on DeepVariant by integrating trio data, requires even greater computational power due to the processing of data from three individuals. When analyzing WGS data at 35x coverage of single human genome on Google Cloud, it typically necessitates a 16-thread CPU instance (Kolesnikov et al., 2021). This tool is optimal for familial studies but can be prohibitive for users without advanced computing setups.

**TABLE 2:** Summary of variant caller computational requirements for a single human genome. ND: not determined.

Tool	Sequencing technology	Hardware support	System Configuration	Runtime (CPU)	Runtime (GPU)	Reported coverage (X)	References
DeepVariant	Illumina	CPU and GPU	CPU: m5.24xlarge 96 threads, GPU: NVIDIA DGX (8 x A100)	5 h	4 min	30	(Accelerate Genomic Analysis for Any Sequencer with NVIDIA Parabricks v4.2 | NVIDIA Technical Blog, 2023)
	PacBio HiFi			5 h	4 min	30	
	ONT			8 h	21 min	55	
DeepTrio	Illumina	CPU and GPU	CPU: n1-standard 16 threads	100 h	ND	35	Kolesnikov et al. (2021)
	PacBio HiFi		ND	ND	ND	ND	
DNAscope	Illumina	CPU only	CPU: 8xlarge 32 threads	30 min	ND	30	Freed et al. (2022a)
	PacBio HiFi		CPU: Intel® Xeon® server 32 threads	3.67 h	ND	30	Freed et al. (2022b)
Clair	Illumina	CPU and GPU	CPU: Intel Xeon Silver 4116 24 threads	1 h	ND	30	Luo et al. (2020)
	PacBio HiFi			1 h	ND	30	
	ONT			5 h	ND	30	
Medaka	ONT	CPU and GPU	CPU: n1-series 16 threads, GPU: 1x NVIDIA Tesla P100	95 h	40 h	50	Shafin et al. (2021)
HELLO	Illumina	CPU and GPU	ND	ND	ND	ND	Ramachandran et al. (2021)
	PacBio		CPU: Intel E5-2683 28 threads	27 min	ND	30 (chr 21)	
HELLO (Hybrid)	Illumina + PacBio	CPU and GPU	ND	ND	ND	ND	
GATK	Illumina	CPU only	CPU: Intel Xeon-P8260 16 threads	44 h	ND	12	Abdelwahab et al. (2023)
	PacBio			102 h		40	
BCFTools	Illumina	CPU only	CPU: Intel Xeon-P8260 16 threads	3 h	ND	12	Abdelwahab et al. (2023)
	PacBio HiFi			39 h		40	
	ONT			8 h		78	

In contrast, DNAscope offers high performance with a lower computational footprint, being designed to operate without GPU acceleration. It can efficiently process Illumina WGS data at 30x coverage in roughly 30 min on a 32-thread CPU system (Freed et al., 2022a), and PacBio HiFi data in about 3.67 h on a 32-core Intel Xeon server (Freed et al., 2022b). DNAscope’s reduced resource requirements, without compromising on accuracy, make it a practical choice for labs balancing performance with computational availability. Clair3 also provides resource efficiency, typically consuming around 7 GB of RAM for Illumina and PacBio data, and requiring about 1 h for 30x coverage WGS processing on two 12-core Intel Xeon Silver 4116 processors (Luo et al., 2020). For ONT data, runtimes extend to around 5 h. Although Clair3 lacks official GPU support, its moderate RAM and CPU needs enable quick processing times, positioning it as an accessible option for labs across various sequencing platforms, especially those engaged with long-read data.

Medaka is specifically tailored for ONT long-read sequencing, and while not as fast as Clair3, it is significantly more efficient than earlier ONT tools like Nanopolish (Jts/nanopolish, 2017) —approximately 50 times faster (Nanoporetech/medaka, 2018). It supports both CPU and GPU, though its computational demands are relatively moderate, favoring ONT projects in labs that lack advanced GPU capabilities. HELLO strikes a balance between computational efficiency and robust performance, requiring around 24 CPU threads and 13 GB of RAM for standard execution, peaking at 29 threads and 18 GB of RAM in optimized flows. Although trained on GPUs, HELLO is optimized for multi-threaded CPU environments, making it accessible without GPU acceleration (Ramachandran et al., 2021). Its efficiency in handling both Illumina and PacBio data makes it suitable for hybrid sequencing projects, where it offers reliable performance without overwhelming computational demands.

In summary, while DeepVariant and DeepTrio offer the highest accuracy, they come with substantial resource requirements, particularly for long-read sequencing data. DNAscope and Clair3 present more resource-efficient alternatives, suitable for labs with limited access to HPC or cloud resources. Medaka and HELLO offer targeted advantages for ONT and hybrid datasets, respectively, while maintaining moderate computational needs, making them accessible for a wider range of research environments.

Notably, the computational resource requirements presented in Table 2 correspond to the analysis of a single human genome at 30x coverage according to literature reports. No normalization was performed to standardize CPU/GPU configurations across the original studies.

Table 3 provides a comparative overview of the AI-based variant callers highlighted in this study. It outlines their support for short-read and long-read sequencing technologies, licensing models (open-source vs. commercial), key advantages and disadvantages, and their ability to handle complex genomic regions such as repetitive or GC-rich areas. Each tool is uniquely suited to specific applications, depending on sequencing platform, computational resources, and project requirements.

**TABLE 3:** Comparative summary of six AI-based variant callers evaluated in this study.

Variant caller	Short-read (Illumina) support	Long-read (PacBio/ONT) support	Open-source vs. Commercial	Advantages	Disadvantages	Handling of complex genomic regions
DeepVariant	Yes	Yes (PacBio HiFi, ONT)	Open-source	High accuracy across platforms, CNN-based architecture, automated filtering	High computational cost, requires GPU for optimal performance	Performs well in repetitive and GC-rich regions
DeepTrio	Yes	Yes (PacBio HiFi)	Open-source	Optimized for trio-based analysis, improved *de novo* mutation detection	High computational cost, requires trio sequencing	Effective in low-coverage and complex regions due to familial context
DNAscope	Yes	Yes (PacBio HiFi, ONT)	Commercial (Sentieon)	High efficiency, optimized for speed and low compute cost	Not fully DL-based, less adaptable to non-human genomes	Performs well in low-depth regions but lacks full CNN adaptability
Clair/Clair3	Yes	Yes (PacBio HiFi, ONT)	Open-source	High performance for long-read sequencing, optimized for low-coverage sequencing	Lacks official GPU acceleration	Strong performance in complex regions and low-quality reads
Medaka	No	Yes (ONT)	Open-source	Optimized for ONT, accurate SNP calling	Lower InDel accuracy, requires high-coverage ONT data	Handles ONT-specific errors effectively
HELLO	Yes	Yes (PacBio)	Open-source	Hybrid caller supporting both Illumina and PacBio, efficient execution	Limited benchmarking on ONT	Performs well with hybrid sequencing data, reduces sequencing errors

## Discussion

The landscape of genomic variant calling is rapidly evolving, driven by the growing adoption of AI-based tools. These tools have demonstrated substantial improvements in accuracy, scalability, and efficiency, addressing many of the challenges posed by complex sequencing technologies and diverse data types (Hall et al., 2024; Junjun et al., 2024). This section examines perspectives, challenges, and future opportunities in this field, while comparing the performance of AI-based tools with conventional methods.

### Perspectives on AI in variant calling

DL models typically require large datasets for training. In the context of AI-based variant callers, all models reviewed in this study were trained using samples from the GIAB dataset. GIAB samples are widely utilized in genomics research for benchmarking and training due to their well-characterized and high-confidence variant annotations. However, the number of training samples and specific methodologies differ across pipelines.

#### Transfer learning capabilities

##### Species transferability

Among the AI-based variant callers, DeepVariant exhibits the highest potential for transfer learning. Studies have demonstrated that a DeepVariant model trained on human genomic data performs better on mouse genomic data than a model trained exclusively on mouse data, indicating effective cross-species generalization (Poplin et al., 2018). Additionally, DeepVariant enables users to train custom models without a gold standard set, using sites consistent with Mendelian inheritance to improve calling accuracy (DeepVariant Blog, 2018). Moreover, DeepVariant allows users to train custom models tailored to specific data types, such as BGISEQ-500 whole genome data (Deepvariant, 2024).

##### Locus transferability

DeepTrio enhances variant calling by leveraging familial relationships, making it more accurate in low-coverage or ambiguous regions. Clair3 and Medaka, trained on long-read data, excel in identifying variants in highly repetitive regions that typically challenge short-read-based tools. HELLO’s hybrid training approach makes it particularly robust when integrating data from different sequencing technologies.

The integration of AI in variant calling brought a major shift in genome analyses. Tools such as DeepVariant, DeepTrio, and Clair3 are examples of how DL architectures can optimize the detection of SNPs and small InDels (Poplin et al., 2018; Huang et al., 2020; Yun et al., 2020; Kolesnikov et al., 2021; Zheng et al., 2022; Brand et al., 2024). These tools go beyond traditional statistical approaches by capturing patterns within sequencing data, offering an enhanced methodology for variant detection (Junjun et al., 2024). For instance, DeepVariant and HELLO consistently achieve SNP F1 scores of 99.9% on Illumina and PacBio HiFi platforms, highlighting their superior performance compared to conventional tools such as GATK (McKenna et al., 2010) and FreeBayes (Garrison and Marth, 2012).

AI-based tools have also facilitated large-scale population studies by enabling efficient data processing and analysis. Projects like the UK Biobank (Szustakowski et al., 2021) and All of Us (Mahmoud et al., 2024) research program have successfully utilized these tools to process large datasets, setting new benchmarks in genomics. Moreover, tools such as HELLO, which integrate hybrid datasets from Illumina and PacBio platforms, demonstrate how AI can address challenges in complex genomic regions by reducing sequencing errors and improving resolution (Ramachandran et al., 2021).

### Challenges in implementing AI variant calling

Despite these advancements, several challenges hinder the broader adoption of AI-based tools. High computational demands are a primary obstacle, particularly for smaller research groups with limited resources. Tools like DeepVariant and DeepTrio rely heavily on GPU resources or HPC environments, and their requirements grow significantly with long-read sequencing data (Kolesnikov et al., 2021; Shafin et al., 2021; Deepvariant, 2023; Brand et al., 2024). While cloud computing offers a viable solution, it raises concerns related to data security, cost scalability, and regulatory compliance, particularly in clinical settings (Minh Dang et al., 2019; Vistro et al., 2020).

While accuracy is a concern for some long-read technologies, several tools, such as Clair3, achieve high InDel detection accuracy on ONT data when optimal sequencing coverage is maintained (Zheng et al., 2022). Coverage plays a critical role in accuracy, as lower coverage can exacerbate sequencing errors and hinder variant calling, especially for challenging genomic regions (Sims et al., 2014; Parks and Lambert, 2015). Addressing these challenges requires refining AI models to adapt to varying sequencing depths and data quality. Additionally, the interpretability of many AI models remains limited (Linardatos et al., 2020; Li X. et al., 2022), raising questions about reproducibility and user confidence, especially in regulatory and diagnostic workflows.

### Superior performance of AI-based tools

Comparative analyses reveal that AI-based variant callers consistently outperform conventional tools in accuracy, sensitivity, and computational efficiency (Abdelwahab et al., 2023). For example, DeepVariant and Clair3 achieve higher detection rates for SNPs and InDels compared to widely used methods like GATK and SAMtools (Li et al., 2009; Danecek et al., 2021), particularly in challenging genomic regions. DeepTrio, with its ability to incorporate familial context, enhances the detection of *de novo* mutations (Kolesnikov et al., 2021; Brand et al., 2024), outperforming non-trio approaches such as Strelka (Saunders et al., 2012; Kim et al., 2018) and FreeBayes (Garrison and Marth, 2012). Similarly, DNAscope’s optimized architecture balances speed and accuracy, making it an ideal choice for clinical applications requiring rapid and precise variant calling (Freed et al., 2022a; Freed et al., 2022b). These advancements underline the transformative potential of AI-based tools to complement or even surpass traditional methods, particularly in high-throughput and precision-driven genomic studies.

### Future opportunities for AI in variant calling

The continued development of AI in variant calling offers numerous opportunities for methodological refinement and broader applications. A key area for improvement lies in the integration of interpretable AI methodologies (Watson, 2022), which would enhance model interpretability and foster greater trust among clinicians and researchers. Additionally, optimizing tools for resource-constrained environments could democratize access to advanced genomic technologies, addressing disparities in global research capabilities.

Further innovation is needed to enhance the robustness and generalizability of AI models through training on diverse datasets (Chen et al., 2023) and sequencing platforms (Yu et al., 2023). Currently, most of these tools were trained with very small and limited dataset such as Genome In A Bottle (GIAB) samples (Genome in a bottle, 2015; Zook et al., 2016). Additionally, hybrid approaches, such as those demonstrated by HELLO, combining data from short-read, long-read, and mixed sequencing platforms, hold great promise for improving variant detection accuracy while mitigating platform-specific limitations. Emerging computational frameworks, including transformer-based architectures and attention mechanisms (Choi and Lee, 2023), could further accelerate processing speeds and improve precision, driving breakthroughs in both research and clinical genomics.

## Conclusion

AI-based tools have significantly advanced the field of variant calling by enhancing accuracy, scalability, and computational efficiency across diverse sequencing platforms. These tools have outperformed conventional methods in key metrics, enabling breakthroughs in both large-scale genomic research and clinical diagnostics. However, challenges such as high computational demands, limited model interpretability, and platform-specific performance constraints remain critical barriers. Addressing these issues will require further methodological innovations, including hybrid data integration and interpretable AI frameworks. Moreover, developing tools optimized for diverse sequencing technologies and resource-constrained environments will be essential for broader adoption. With continued advancements, AI-driven variant callers have the potential to redefine genomic research and enable transformative applications in personalized medicine, population studies, and beyond.
